# Focal laser photocoagulation for circumscribed choroidal hemangioma: a case report

**DOI:** 10.1097/RC9.0000000000000907

**Published:** 2026-09-01

**Authors:** Badr Alahmadi, Alaa Almuzaini, Razan Alawaz, Esraa Alshareef, Ali Almalki

**Affiliations:** a Ophthalmology Department, Ministry of National Guard Health Affairs, Madina, Saudi Arabia; b Pediatric Ophthalmology and Strabismus Division, King Khaled Eye Specialist Hospital, Riyadh, Saudi Arabia

**Keywords:** anti-VEGF, choroidal hemangioma, choroidal tumors, focal laser photocoagulation, subretinal fluid

## Abstract

**Background:**

Circumscribed choroidal hemangioma (CCH) is a benign vascular tumor that may cause visual loss due to chronic subretinal fluid and macular involvement. Various treatment modalities have been described, including photodynamic therapy, laser photocoagulation, and anti-vascular endothelial growth factor (VEGF) therapy. We report a case of symptomatic CCH successfully treated with focal laser photocoagulation after an inadequate response to intravitreal aflibercept.

**Methods:**

A 59-year-old male presented with progressive visual decline in the left eye. Clinical examination and multimodal imaging, including fundus photography, optical coherence tomography (OCT), and fluorescein angiography (FA), were performed. The patient initially received three monthly intravitreal aflibercept injections without significant anatomical or functional improvement. Focal laser photocoagulation was subsequently applied directly over the extrafoveal lesion.

**Results:**

Fundus examination revealed an orange choroidal lesion nasal to the optic disc, with associated subretinal fluid extending to the macula. OCT demonstrated subretinal and intraretinal fluid, while FA showed progressive hyperfluorescence consistent with CCH. Following two sessions of focal laser photocoagulation, complete resolution of subretinal fluid was achieved. Best-corrected visual acuity improved from 6/120 to 6/15 and remained stable throughout 18 months of follow-up without recurrence.

**Conclusion:**

Focal laser photocoagulation can provide favorable anatomical and visual outcomes in selected patients with extrafoveal CCH, particularly when anti-VEGF therapy is ineffective and photodynamic therapy is unavailable.

## Introduction

Circumscribed choroidal hemangioma (CCH) is an uncommon, noncancerous growth characterized by vascular tissue formation that develops in the choroid layer of the eye^[^1,2^]^. It is considered congenital; it often does not cause symptoms until the fourth to sixth decades of life^[^3^]^.

CCH is composed of dilated choroidal vessels but does not involve the choriocapillaris^[^4^]^. The underlying cause of visual loss is transudative leakage, with resultant retinal edema and macular detachment^[^3^]^. Treatment strategies have included laser photocoagulation, transpupillary thermotherapy (TTT), photodynamic therapy (PDT), radiotherapy, and anti-VEGF therapy; the optimal management depends on tumor location, associated exudation, and resource availability^[^3^]^. Limitations of treatment have included recurrent subretinal fluid and complications of treatment, such as foveal damage resulting from the treatment of macular tumors, eventually leading to permanent vision loss.

HIGHLIGHTS

- Circumscribed choroidal hemangioma can cause significant visual loss due to chronic subretinal fluid.
- Anti-VEGF therapy may be ineffective in CCH due to its non–VEGF-driven vascular pathology.
- Focal laser photocoagulation achieved complete resolution of subretinal fluid in this case.
- Focal laser remains a practical and effective option for extrafoveal CCH, especially when PDT is unavailable.

We report a case of symptomatic extrafoveal CCH that was successfully treated with focal laser photocoagulation following an inadequate response to intravitreal aflibercept.

This case report is submitted in line with the SCARE Guidelines checklist^[^5^]^.

## Case description

A 59-year-old male presented to our clinic in February 2023 with a history of a gradual decrease in vision in the left eye (OS) over 5 years, which had worsened over the preceding 2 years. He had been followed at another hospital during that period without intervention. His medical history was significant for hypertension and dyslipidemia, both controlled with medication. The patient reported no relevant family history of ocular tumors or hereditary ocular disorders.

On examination, the best-corrected visual acuity (BCVA) was 6/8.5 in the right eye (OD) and 6/120 in the left eye (OS). Intraocular pressures were within normal limits in both eyes. Anterior segment examination was unremarkable bilaterally.

Fundus examination of the right eye was normal. Examination of the left eye revealed a slightly elevated, orange-colored choroidal lesion located approximately one disc diameter nasal to the optic disc, associated with overlying retinal pigment epithelium (RPE) changes. The macula showed loss of the foveal reflex, with a shallow serous neurosensory detachment and yellowish subretinal deposits at the lesion margins (Fig. 1A).

**Figure 1.**
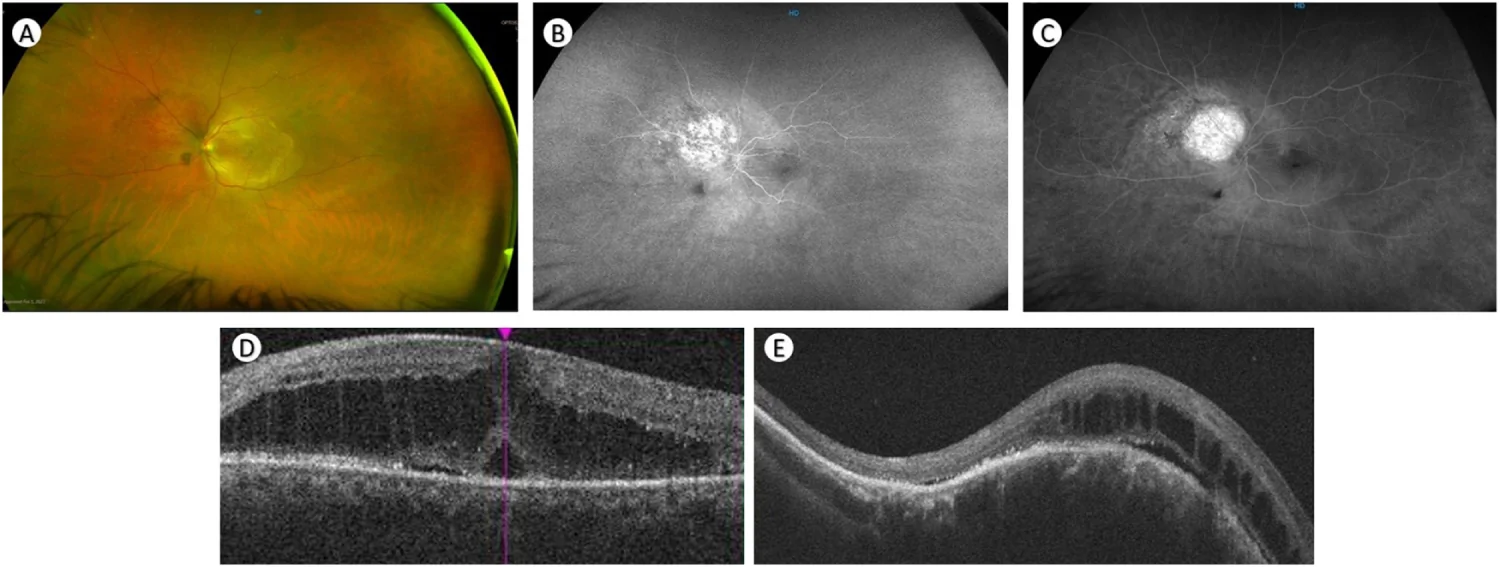
Baseline multimodal imaging of the left eye. (A) Color fundus photograph of the left eye showing an elevated choroidal lesion nasal to the disc, with an orange hue and associated RPE changes. The macula demonstrates loss of the foveal reflex and a shallow serous neurosensory detachment with yellowish, feathered-edged subretinal material. (B) Baseline optical coherence tomography (OCT) of the macula demonstrating subretinal and intraretinal fluid. (C) OCT over the lesion showing subretinal fluid and retinal elevation. (D) Early-phase fluorescein angiography showing spotty hyperfluorescence. (E) Late-phase fluorescein angiography showing diffuse leakage.

Optical coherence tomography (OCT) demonstrated subretinal fluid involving both the lesion and the macula, intraretinal cystic changes, and an epiretinal membrane (Fig. 1B and C). Fluorescein angiography (FA) revealed early spotty hyperfluorescence in the arterial phase, with late diffuse leakage (Fig. 1D and E). B-scan ultrasonography demonstrated an acoustically solid choroidal mass with high internal reflectivity. Based on these findings, a diagnosis of a left nasal CCH with subretinal fluid extending to the fovea was made.

The differential diagnosis included amelanotic choroidal melanoma, choroidal metastasis, and chronic central serous chorioretinopathy. However, the characteristic orange coloration, high internal reflectivity on B-scan ultrasonography, absence of low-to-medium internal reflectivity typically seen in melanoma, and the angiographic pattern of progressive, diffuse hyperfluorescence supported the diagnosis of CCH. In addition, the lesion showed no features suggestive of metastatic disease, such as multifocality or a creamy-yellow appearance.

Because PDT was not readily available at our center, an initial trial of intravitreal anti-VEGF therapy was attempted as a minimally invasive option to reduce vascular permeability, subretinal fluid, and associated macular edema before proceeding to more invasive focal treatment. The patient therefore received three monthly intravitreal aflibercept injections. However, no significant anatomical or visual improvement was observed.

Therefore, **in May 2023**, focal laser photocoagulation was performed directly over the extrafoveal lesion, which was located approximately one disc diameter nasal to the optic disc. Treatment parameters included a spot size of 100 μm, a power of 200 mW, and a duration of 0.3 seconds. A total of 215 laser spots were applied to the lesion.

**In June 2023**, the patient reported subjective improvement in color perception. Although visual acuity remained unchanged, OCT demonstrated a reduction in subretinal and intraretinal fluid, and an additional laser augmentation session was planned (Fig. 2A).

**Figure 2.**
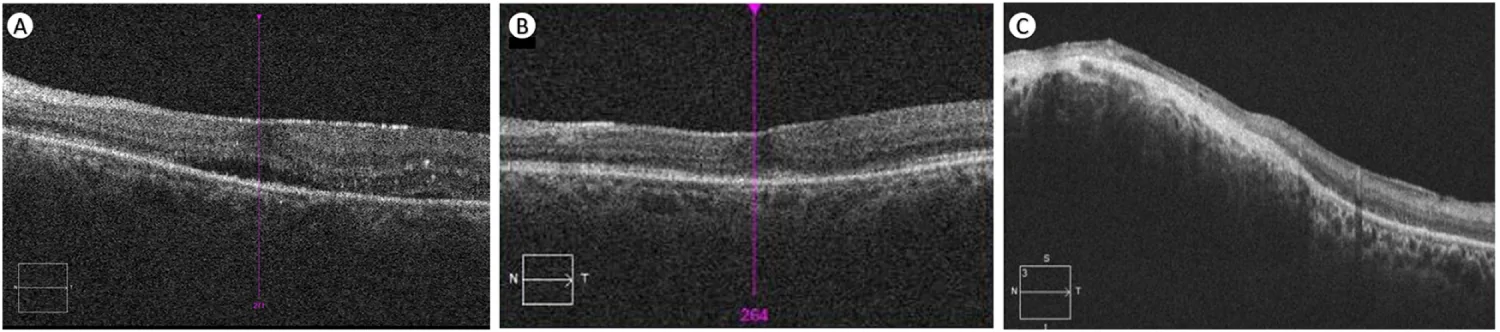
Response to focal laser photocoagulation. (A) OCT obtained after the first laser session, demonstrating an interval reduction in subretinal and intraretinal fluid. (B,C) OCT obtained after the second laser session, showing complete resolution of the subretinal and intraretinal fluid, with a residual epiretinal membrane in the macular area and overlying the lesion.

**By July 2023**, BCVA improved from 6/120 to 6/40 in the left eye. OCT demonstrated complete resolution of subretinal fluid, with minimal residual intraretinal fluid and a persistent epiretinal membrane, after which additional focal laser augmentation was performed (Fig. 2B and C).

**In August 2023**, BCVA remained stable at 6/40. OCT confirmed complete resolution of subretinal fluid at both the lesion and the macula, with residual epiretinal membrane and focal outer retinal layer loss (Fig. 3A). FA demonstrated minimal residual leakage (Fig. 3B). The patient was subsequently observed.

**Figure 3.**
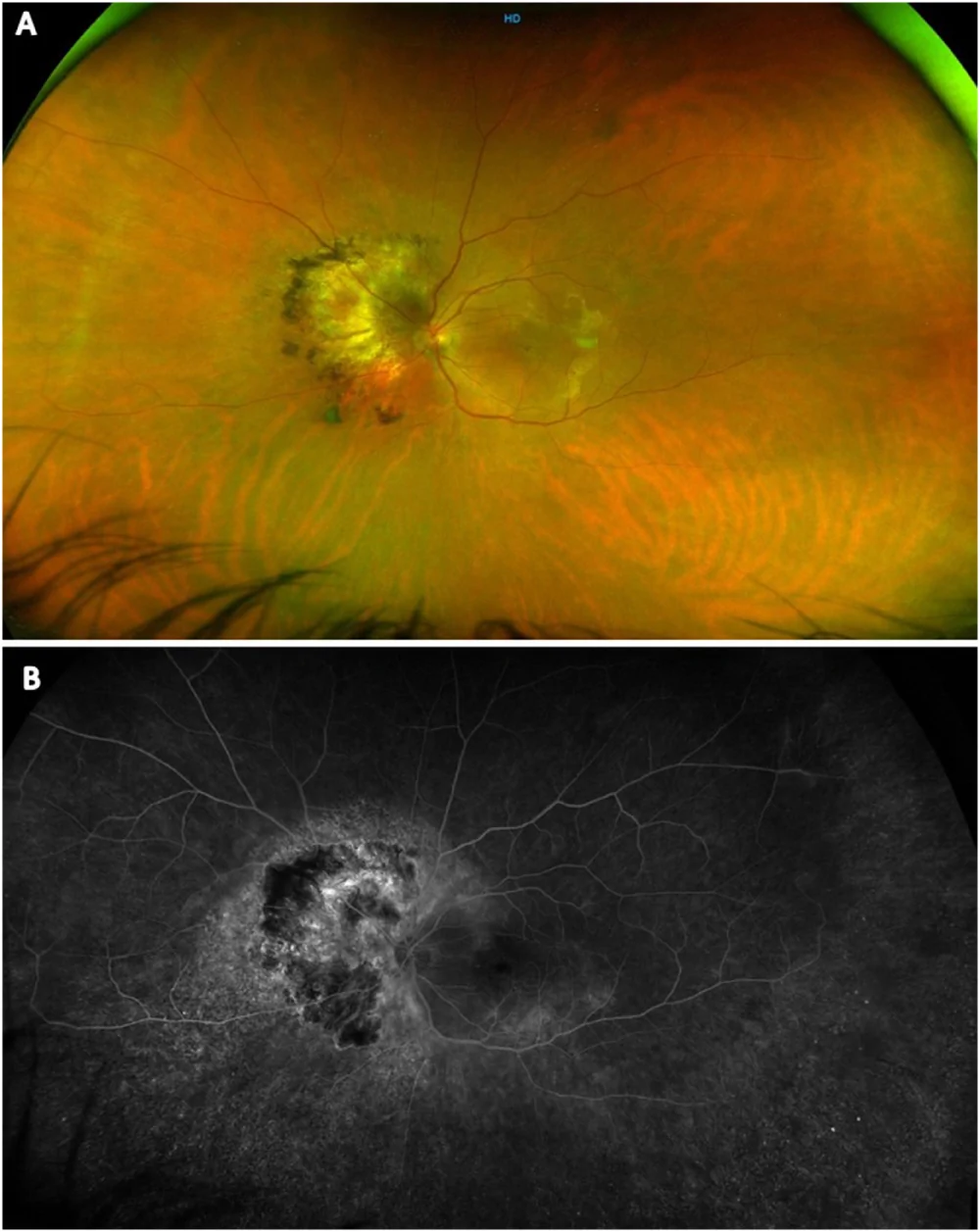
Final outcome following treatment. (A) Color fundus photograph demonstrating a well-defined chorioretinal scar at the treatment site, with restoration of the foveal contour. (B) Final fluorescein angiography showing minimal residual leakage and a stable post-treatment appearance.

At the most recent follow-up in **February 2025**, the BCVA in the left eye improved to 6/15 with a refraction of +2.00 − 0.50 × 105. Clinical examination, OCT, and FA findings remained stable, without recurrence of subretinal fluid or evidence of tumor activity.

## Discussion

CCH is a benign vascular tumor of the choroid that may cause significant visual loss due to chronic subretinal fluid, macular edema, or secondary retinal RPE changes. The decision to treat depends mainly on the tumor’s location, size, and the presence of visual symptoms. As reported by Shields *et al*, in a large series of 200 patients, the most common cause of decreased visual acuity in CCH was subretinal fluid and chronic macular edema rather than tumor size itself^[4]^.

The diagnosis of CCH is primarily clinical and supported by multimodal imaging. In the present case, the characteristic orange-red appearance, high internal reflectivity on B-scan ultrasonography, and progressive diffuse hyperfluorescence on FA favored the diagnosis of CCH and helped to exclude malignant choroidal lesions.

The primary aim of therapy is to reduce vascular leakage and resolve the subretinal fluid to restore or preserve vision. Tumor shrinkage is considered a secondary goal. Various therapeutic modalities have been proposed, including laser photocoagulation, TTT, PDT, external beam radiotherapy, plaque brachytherapy, and anti-VEGF injections. The choice of treatment depends on the lesion’s location and the availability of resources.

PDT is currently considered the preferred treatment modality in many centers because of its selective vascular occlusion and favorable safety profile. PDT achieves high rates of tumor control and resolution of exudative retinal detachment while minimizing damage to the surrounding retina. Reported tumor control rates range from 73% to 100%, with improvement of at least two lines of visual acuity in most treated patients and complete resolution of macular exudation in many cases^[^6–8^]^. However, the availability and cost of PDT may limit its use in some institutions and resource-limited settings.

In the present case, PDT was not readily available; therefore, intravitreal aflibercept was initially selected because of its availability and its potential to temporarily reduce vascular permeability, subretinal fluid, and associated macular edema in a minimally invasive manner before proceeding to focal laser treatment. However, despite three monthly injections, no significant anatomical or functional improvement was observed. This limited response is consistent with the underlying pathophysiology of CCH, which represents a low-grade vascular malformation rather than a VEGF-driven neoplasm. Although anti-VEGF agents may temporarily reduce subretinal fluid in selected cases, their role as monotherapy remains limited and inconsistent. Sagong *et al*^[^9^]^ reported improvement in visual acuity and reduction of retinal fluid in cases treated with bevacizumab, either following recurrence after laser photocoagulation or in combination with PDT. Nevertheless, the overall efficacy of anti-VEGF monotherapy in CCH remains uncertain^[^9^]^.

Following failure of anti-VEGF therapy, focal laser photocoagulation was performed directly over the extrafoveal lesion, resulting in complete resolution of subretinal fluid and sustained visual improvement from 6/120 to 6/15 over long-term follow-up. Laser photocoagulation remains one of the earliest and most accessible treatment options for extrafoveal CCH. It induces thermal occlusion of abnormal choroidal vasculature, thereby reducing vascular leakage and promoting reabsorption of subretinal fluid. Smith and Johnson reported resolution of subretinal fluid in 62%–100% of cases treated with scatter laser photocoagulation, although tumor size reduction was limited^[^10^]^. Anand *et al* reported recurrence of subretinal fluid in approximately 40% of treated cases^[^11^]^.

Nevertheless, focal laser therapy has recognized limitations. Repeated sessions may result in chorioretinal scarring, retinal pigment epithelial atrophy, visual field defects, or secondary choroidal neovascularization^[^6^]^. Therefore, laser treatment is generally best suited for extrafoveal lesions where the risk of central retinal damage is minimized.

The present case highlights a favorable long-term anatomical and functional outcome following focal laser photocoagulation after an inadequate response to anti-VEGF therapy. Although PDT remains the standard of care in many centers, focal laser photocoagulation may still represent a practical, safe, and cost-effective alternative in selected extrafoveal cases, particularly in resource-limited settings or when PDT is unavailable.

## Conclusion

This case demonstrates that focal laser photocoagulation can achieve good anatomical and visual outcomes in patients with CCH. It is a practical and cost-effective option, particularly in resource-limited settings or when more advanced therapies such as PDT are unavailable. Regular follow-up is important to monitor for recurrence or complications.

## Data Availability

All data relevant to this case are included in the article. Further information is available from the corresponding author upon reasonable request.
